# Engineering a Disulfide Bond in the Lid Hinge Region of *Rhizopus chinensis* Lipase: Increased Thermostability and Altered Acyl Chain Length Specificity

**DOI:** 10.1371/journal.pone.0046388

**Published:** 2012-10-02

**Authors:** Xiao-Wei Yu, Nian-Jiang Tan, Rong Xiao, Yan Xu

**Affiliations:** 1 State Key Laboratory of Food Science and Technology, Jiangnan University, Wuxi, China; 2 Key Laboratory of Industrial Biotechnology, Ministry of Education, School of Biotechnology, Jiangnan University, Wuxi, China; 3 Center for Advanced Biotechnology and Medicine, Department of Molecular Biology and Biochemistry, Rutgers University, Piscataway, New Jersey, United States of America; Russian Academy of Sciences, Institute for Biological Instrumentation, Russian Federation

## Abstract

The key to enzyme function is the maintenance of an appropriate balance between molecular stability and structural flexibility. The lid domain which is very important for “interfacial activation” is the most flexible part in the lipase structure. In this work, rational design was applied to explore the relationship between lid rigidity and lipase activity by introducing a disulfide bond in the hinge region of the lid, in the hope of improving the thermostability of *R. chinensis* lipase through stabilization of the lid domain without interfering with its catalytic performance. A disulfide bridge between F95C and F214C was introduced into the lipase from *R. chinensis* in the hinge region of the lid according to the prediction of the “Disulfide by Design” algorithm. The disulfide variant showed substantially improved thermostability with an eleven-fold increase in the *t*_1/2_ value at 60°C and a 7°C increase of *T*_m_ compared with the parent enzyme, probably contributed by the stabilization of the geometric structure of the lid region. The additional disulfide bond did not interfere with the catalytic rate (*k*_cat_) and the catalytic efficiency towards the short-chain fatty acid substrate, however, the catalytic efficiency of the disulfide variant towards pNPP decreased by 1.5-fold probably due to the block of the hydrophobic substrate channel by the disulfide bond. Furthermore, in the synthesis of fatty acid methyl esters, the maximum conversion rate by RCLCYS reached 95% which was 9% higher than that by RCL. This is the first report on improving the thermostability of the lipase from *R. chinensis* by introduction of a disulfide bond in the lid hinge region without compromising the catalytic rate.

## Introduction

Lipases (triacylglycerol ester hydrolases EC 3.1.1.3) are one of the most versatile industrial enzymes for their specificity in hydrolysis, interesterification, alcoholysis, acidolysis, esterification and aminolysis, preferentially at the interface between lipid and water in heterogeneous systems [1]. Lipases are widely used in various industries, such as the detergent, food, bioenergy, flavour industry, biocatalytic resolution of pharmaceuticals, esters and amino acid derivatives, making of fine chemicals, agrochemicals [2].

Thermostability proved to be a key factor among the desirable characteristics that commercially important lipases should exhibit, since the role of enzymes in many processes has been known for a long time [3]. To meet this end, rational design [4] and directed evolution [5] methods have been used extensively to improve stability of lipases for challenging industrial applications.

Disulfide bridges are believed to stabilize proteins mostly through an entropic effect, by decreasing the entropy of the protein's unfolded state [6]. The stabilizing effect of disulfide bridges is confirmed by many mutagenesis studies involving the introduction of disulfide bonds in enzymes [7]. For example, the thermostability of *Rhizomucor miehei* lipase was improved with half-life approximately fivefold than that of native enzyme at 60°C by introducing a disulfide bond between P96C and K106C [8]. The introduction of a disulfide bond into the neutral protease from *Bacillus stearothermophilus* by the double mutations G8C/N60C had resulted in an extremely thermostable enzyme with a half-life of 35.9 min at 92.5°C [9]. The thermostability of *Thermomyces lanuginosus* GH11 xylanase was improved significantly by engineering disulfide bridge Q1C/Q24C with 20-fold increase of the half-life at pH 8 and 70°C [10]. On the other hand, the introduction by protein engineering of a disulfide bond could also bring to detrimental effects on protein stability by influence of the global stability of the enzyme [11]–[13]. The extra disulfide bond bridging domains A and B of α-amylase globally stabilized the mutant according to the calorimetric studies. However, the strain imposed on the active site architecture induces a 2-fold higher thermal inactivation rate at 45°C as well as the appearance of a less stable calorimetric domain. It supports the subtle variations that a cross-link can induce in an enzyme or a protein in general to allow specific environmental adaptations [14]. The thermodynamics of denaturation of barnase disulfide mutants showed that the subtle balance of intramolecular and solvation contributions depend on the specific site of the disulfide bonds occupied within the overall structure of the protein [15].

In our previous study, *Rhizopus chinensis* CCTCC M201021 screened from Daqu of brewing strong aromatic Chinese spirits showed a high potential for industrial usage, including synthesis of eicosapentaenoic acid (EPA), docosahexaenoic acid (DHA). sorbitan oleate and ethyl esters [16]–[20]. And the lipase gene *proRCL* from *R. chinensis* was cloned from this strain and was expressed at high-level in *Pichia pastoris* which was about 580 times higher than that of the wild-type *R. chinensis* lipase (RCL) [21]. However, the use of the lipases from *Rhizopus* sp. in industrial applications is restricted by their low thermostability.

Most lipases, including RCL, have a lid domain that covers its catalytic triad and the movement of an α-helical lid by rotating around two hinge regions at the lipid-water interface created a large hydrophobic patch around the catalytic triad, resulting in activation of the lipase [22]–[24]. It is interesting to note that the functional important lid domain is also one of the most flexible parts in the structure of lipases. The key to protein function is the maintenance of an appropriate balance between molecular stability on the one hand and structural flexibility on the other. Stability is needed to ensure the appropriate geometry for ligand binding, as well as to avoid denaturation, while flexibility is necessary to allow catalysis at a metabolically appropriate rate [25]. Thus, the question raised is how to stabilize the lid domain while at the same time to maintain its function for “interfacial activation”. In the current study, a rational design was applied to explore the relationship between lid rigidity and lipase activity by introducing a disulfide bond in the hinge region of the lid, in the hope of improving the thermostability of *R. chinensis* lipase without compromising its catalytic performance. And the thermostable lipase was subjected to transesterification of fatty acid methyl esters.

## Results and Discussion

### Design of the disulfide variants

Disulfide by Design is an program for the rational design of disulfide bonds in proteins based on algorithms created for disulfide identification in protein fold recognition methods [26]. For a given protein structural model, all residue pairs are rapidly assessed for proximity and geometry consistent with disulfide formation, assuming the residues were mutated to cysteines. The output displays residue pairs meeting the appropriate criteria. The Disulfide by Design algorithm has been successfully used for disulfide engineering [8], [27], [28].

**Figure 1:**
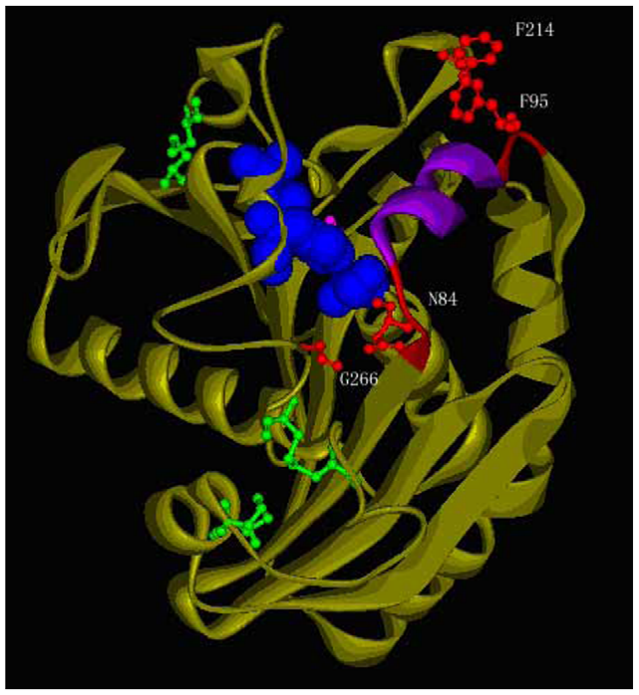
Models of the simulated structure of RCL in a close form. Red, the lid hinge regions (^82^GTNS^85^ and ^92^DMVFT^96^), F214/F95 and N84/G266 as ball and stick; Blue, the catalytic triad of S145-H257-D204; Green, three original disulfide bonds; Pink, the free cysteine C177; Purple, the lid region (^86^FRSAIT^91^).

We aimed at increasing the stability of *R. chinensis* lipase by introducing a disulfide bond in the hinge region of the lid. Fig. 1 shows the three-dimensional model of *R. chinensis* lipase built by SWISS-MODEL. The structure model showed the highest homologous to the structure of *R. niveus* lipase (RNL) at 2.2 Å resolution (lLGY) with 81% similarity. Amino acid sequence alignment of the two mature lipases showed that the identity between RNL and RCL (GenBank Accession No. EF405962) was 82.16% (Fig. S1). The secondary structure of *R. chinensis* lipase consists of nine *a* -helices and eight β-strands. Three disulfide bonds (Cysteines 29–268, 40–43, and 235–244) stabilize the molecule and one free cysteine (C177) exists in the molecule. The lid domain of *R. chinensis* lipase consists of six amino acids (underlined, ^82^GTNSFRSAITDMVFT^96^) plus 4∼5 amino acids as two hinges around the lid. The software Disulfide by Design was effective in predicting the residue pairs to form disulfide bonds which gave 34 residue pairs satisfying the geometric constraints for disulfide bonds, including the three original disulfide bonds. Disulfide bonds are selected in the principle that they have relatively low bond energy with Chi3 (χ3 torsion angle) value between ±90 and ±110, which may be desirable to form disulfide bonds. Chi3 is formed by the Cβ-Sγ-Sγ-Cβ bonds, with rotation about the Sγ-Sγ bond. Two disulfide bonds each with one substitution located in the lid hinge region were selected, which were F95C/F214C (RCLCYS) and N84C/G266C (RCLCYSN).

### Expression and structure analysis of RCL and disulfide variants

RCLCYS and RCLCYSN were constructed and expressed in *P. pastoris*. The secondary structure determined by CD spectroscopy in the “far-UV” spectral region (190–250 nm) (data not shown) suggested that RCLCYS folded properly as the native lipase, while RCLCYSN aggregated and showed quite different CD spectrum and low activity. The simulated 3-D model shows that F95C located at the end of the lid hinge region, and F214C located on a separately loop (Fig. 1), between which the disulfide bond could be formed to stabilize the geometric structure of the lid and the loop after substitution. Moreover, the positions of the amino acids are far away from the catalytic center of *R. chinensis* lipase (S145:OG-F95:N, 15.2 Å) and the free cysteine177 (C177:N-F95:N, 16.6 Å) (Fig. 1), avoiding the disturbance of the catalytic activity and the mismatch of the disulfide bond. The other pair of disulfide bond (N84C and G266C) exhibited a negative effect on the protein structure. N84C located at the other side of the lid region was predicted to form disulfide bond with G266C at the C terminal. N84C seemed to be too close to the lid region (N84:N-F86:N, 6.3 Å; compared with F95:N-T91:N, 10.3 Å), which may interfere with the opening of the lid domain.

**Figure 2:**
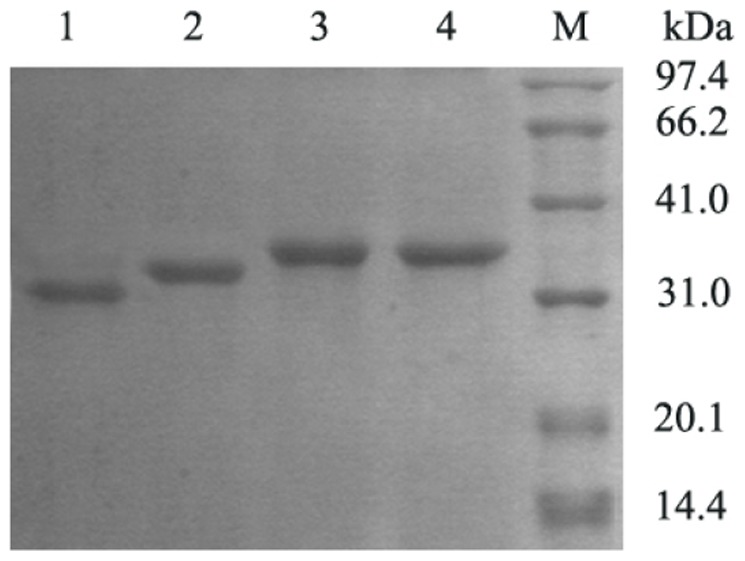
SDS-PAGE analysis of RCLCYS and RCL under reducing and nonreducing conditions. Lane 1 nonreduced RCLCYS, Line 2 nonreduced RCL, Line 3 reduced RCLCYS, Line 4 reduced RCL.

Since RCLCYSN showed very low activity, this variant was not analyzed further. The presence of disulfide bonds of RCLCYS and RCL was analyzed by comparing mobilities during SDS-PAGE of enzyme samples that had been prepared in the absence or the presence of reducing reagent (Fig. 2). Nonreducing SDS-PAGE showed that the double mutant enzyme migrated slightly faster than the wild-type enzyme, whereas identical mobilities were observed in the presence of reducing reagent DTT. Because in the presence of high concentration of DTT, RCL containing three disulfide bonds and RCLCYS containing four disulfide bonds were reduced completely which result in identical conformations. Nevertheless, under the nonreducing condition the electrophoretic mobility of the more compact form of RCLCYS should be faster due to less retardment from gel matrix.

### Thermostabilities of RCLCYS and RCL

**Figure 3:**
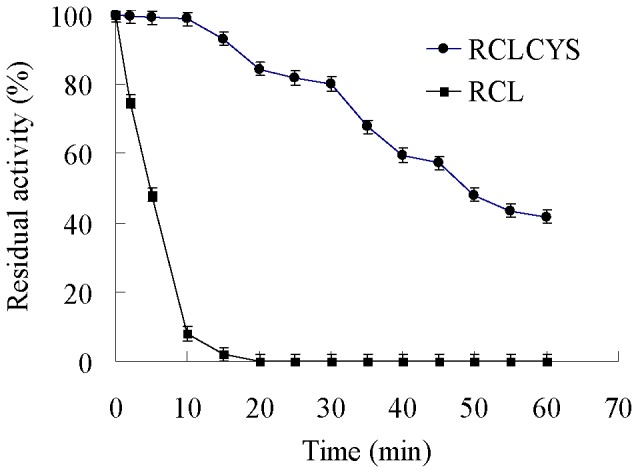
Thermal inactivation of RCLCYS and RCL at 60°C.

Purified, electrophoretically homogeneous wild-type and mutant enzyme were used for determining half-life *t*_1/2_ at 60°C. As shown in Fig. 3, the mutant RCLCYS was considerably more stable than the wild-type enzyme. The wild-type lipase RCL showed significant loss of enzyme activity at 60°C, by almost 50% at 4 min, and after 10 min, the enzyme activity dropped to about 95% of the original, while the disulfide mutant showed substantially improved enzyme stability due to the formation of the new disulfide bond, with the increase of the half-life to 46 min at 60°C.

**Figure 4:**
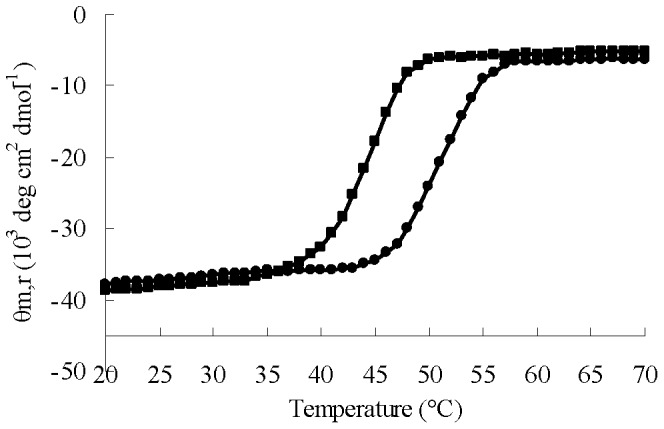
Heat-induced denaturation of RCLCYS and RCL. CD spectra of RCLCYS (•) and RCL (▪) were recorded at 220 nm at the temperatures indicated.

To determine the stability of the mutant RCLCYS, its heat-induced denaturation was monitored by CD spectroscopy at 220 nm (Fig. 4). The denaturation of the parent lipase RCL followed a sigmoidal curve with a transition at 45°C, and the folding intermediate of RCLCYS denatured at 52°C (Fig. 4), indicating that RCLCYS was more stable than the parent lipase to heat-induced denaturation.

### Enzyme Properties of RCLCYS and RCL

**Figure 5:**
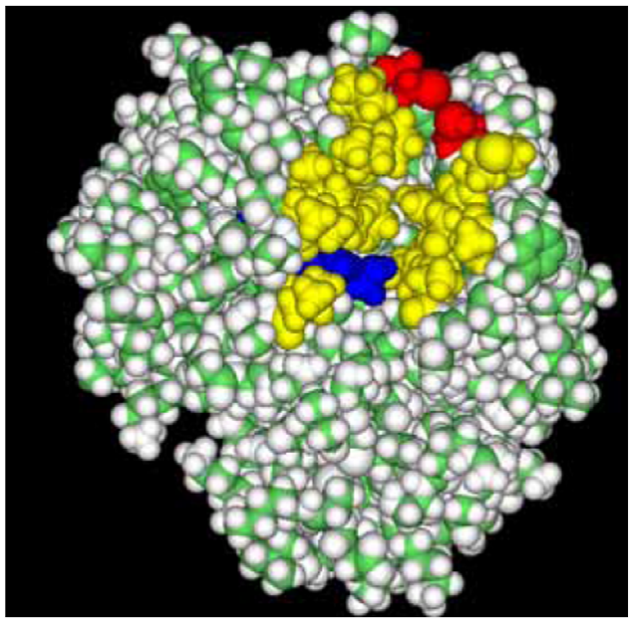
Proposed substrate binding region of RCLCYS in an open form. Red, the introduced disulfide bond; Blue, the catalytic triad of S145-H257-D204; Yellow, the substrate binding region.

As shown in Table 1, the substitution in the mutant enzyme had no effect on the optimum pH (8.5) as well as the optimum temperature (40°C) compared with these of wild-type enzyme. RCLCYS showed altered kinetic parameters. With pNPA (*p*-nitrophenyl acetate, C2:0) as substrate, both the *K*_m_ and *k*_cat_ value of RCLCYS show little difference compared with these of RCL. However, with pNPP (*p-*nitrophenyl palmitate, C16:0) as substrate, the *K*_m_ value of RCLCYS was almost 1.5 times higher than that of RCL with almost the same *k*_cat_ value. According to the catalytic efficiency values of *k*_cat_/*K*_m_, it suggested that RCLCYS preferred the short-chain fatty acid substrate (pNPA, C2:0) while RCL preferred the long-chain fatty acid substrate (pNPP, C16:0). The results could be interpreted by the three dimension model of RCLCYS. In Fig. 5, the yellow part of the structure of the lid open form indicated the substrate binding region according to the reports of *R. delemar* lipase [29] and *R. oryzae* lipase [30]. The substrate binding region of RCL consists of the amino acids T83, A89, D92, M93 (I93 in RNL), F95, F112, L146, P178, I205, V206, V209, P210, P211, F214, I254 (L254 in RNL), L258, which are well conserved in *Rhizopus* sp. lipases. The introduced disulfide bond was located at the end of the binding domain, which might interfere the binding of the long- chain fatty acid substrate and thus, lower the affinity of the long-chain fatty acid substrate for RCLCYS. Klein *et al.* [31] constructed a double mutant F95D/F214R of *R. delemar* lipase at the same conserved position as the *R. chinensis* lipase. Molecular models of the double mutant suggest the possibility of a salt bridge between D95 and R214 across the distal end of the acyl binding groove, which also resulted in the decreased selectivity towards long-chain fatty acid substrate. However, the thermostability of the double mutant with an additional salt bridge was not investigated.

**Table 1:** Enzyme properties of RCLCYS and RCL.

Property	RCL	RCLCYS
Optimum temperature (°C)	40	40
Optimum pH	8.5	8.5
*K*_m_ (µM, Substrate pNPA)	295±6	298±8
*k*_cat_ (s^−1^, Substrate pNPA)	17.7±0.2	17.3±0.2
*k*_cat_/*K*_m_ (M^−1^ s^−1^, Substrate pNPA)	6.00*10^4^	5.80*10^4^
*K*_m_ (µM, Substrate pNPP)	304±7	450±6
*k*_cat_ (s^−1^, Substrate pNPP)	18.9±0.4	18.4±0.2
*k*_cat_/*K*_m_ (M^−1^ s^−1^, Substrate pNPP)	6.22*10^4^	4.09*10^4^

### Biosynthesis of fatty acid methyl esters

**Figure 6:**
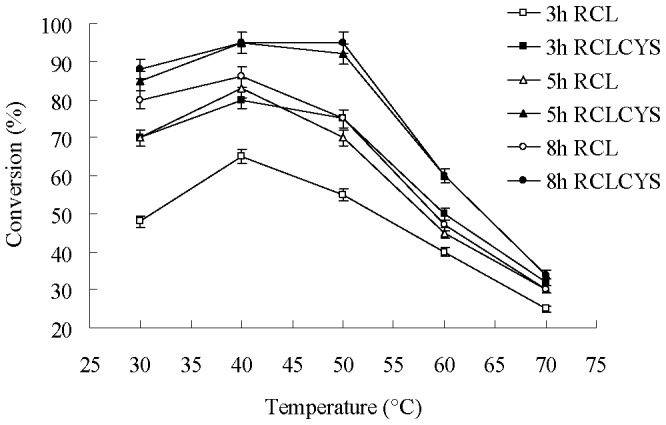
Solvent-free conversion of soy bean oil to fatty acid methyl esters by RCLCYS and RCL at different temperatures. The various curves refer to the conversion of soy bean oil by lipases as a function of temperature at different intervals of reaction time.

In our previous study, the whole cell lipase from *R. chinensis* CCTCC M201021 showed high catalytic ability in the biocatalysis of soy bean oil and methanol in solvent-free conditions [20]. Here, we employed the free *R. chinensis* lipase and the disulfide mutant to synthesize fatty acid methyl esters. Firstly, we investigated the effect of temperature on the transesterification reaction. Reaction rate increased with temperature, however this process does not continue indefinitely. Indeed, enzyme inactivation occurs above a certain temperature and the catalytic activity decreases. Fig. 6 reports the conversion of soy bean oil by RCLCYS as a function of temperature at different intervals of reaction time compared with the reaction by RCL. The conversion trends at different temperatures were as expected. The conversions by both lipases increased up to the temperature of 40°C in consistent with the optimal temperature and decreased due to a further temperature enhancement before 5 h. After 8 h the conversion by the thermostable RCLCYS reached 95% at 40°C, remained constant up to 50°C and decreased at 60°C. However, at this time point the conversion by the wild-type lipase RCL reached to maximum (86%) at 40°C and the conversion decreased to 75% at 50°C. It suggested that the introduced disulfide bond increased the thermostability of the lipase RCLCYS which obtained a higher conversion and showed better performance at higher temperature.

**Figure 7:**
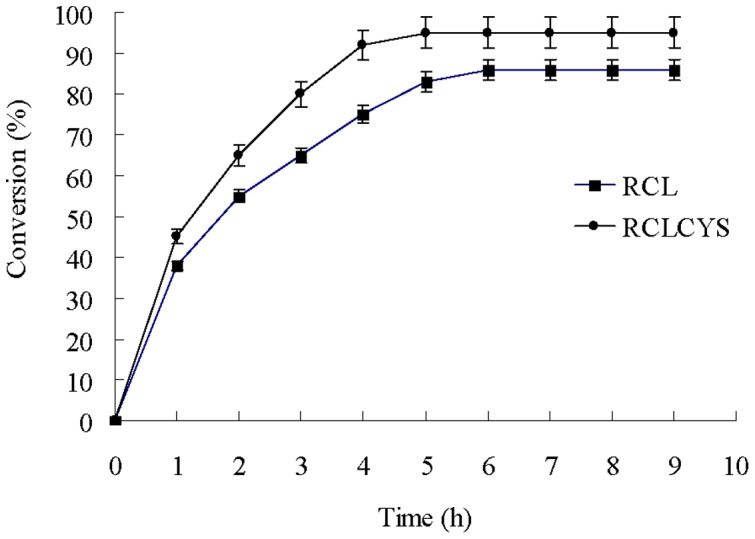
Time course of solvent-free conversion of soy bean oil to fatty acid methyl esters by RCLCYS and RCL at 40°C.

Then, the synthesis of fatty acid methyl esters was studied at 40°C. As shown in Fig. 7, the maximum conversion rate by RCLCYS reached 95% after 4 h which was 9% higher than that by RCL. After 1 h, the conversion rate by RCL decreased a little more quickly than that by RCLCYS which indicated that the disulfide variant could maintain better activity contributed by the improved thermostability compared to the wild-type lipase.

## Conclusions

A disulfide bridge between F95C and F214C was introduced into the lipase from *R. chinensis* in the hinge region of the lid according to the prediction of the “Disulfide by Design” algorithm and the 3-D structure model. The disulfide mutant showed substantially improved thermostability with an eleven-fold increase in the *t*_1/2_ value at 60°C and a 7°C increase of *T*_m_ compared with the parent enzyme, probably contributed by the stabilization of the geometric structure of the lid region. The investigation of enzymatic properties of the disulfide variant revealed that the additional disulfide bond did not interfere with the catalytic rate (*k*_cat_) and the catalytic efficiency towards the short-chain fatty acid substrate, however, the catalytic efficiency of the disulfide variant towards pNPP decreased by 1.5-fold by blocking the hydrophobic substrate channel. It suggests that introduction of the disulfide bond in the lid hinge region restricted the movement of the lid domain to some extent resulting altered catalytic properties. Furthermore, in the synthesis of fatty acid methyl esters, the thermostable variant showed a conversion of 95% and better performance at higher temperature compared with these of the wild-type lipase, indicating the potential in industry. This is the first report on improving the thermostability of the lipase from *R. chinensis* by introduction of a disulfide bond in the lid hinge region in the purpose to stabilize the lid domain without compromising the catalytic rate. The results clearly demonstrated the advantage of knowledge-based rational design by employing the information derived from the 3-D structure model. The strategy is expected to be extended to the engineering of other thermostable lipases.

## Materials and Methods

### Enzyme and chemicals

Restriction enzyme, T4 DNA ligase, *Taq* DNA polymerase, PCR reagent (TaKaRa Biotechnology (Dalian) Co., Ltd.), primers (SBS Gene Technology (Shanghai) Co., Ltd.), Gel Extraction Kit, PCR Purification Kit (Bioflux), Plasmid Mini Kit I (OMEGA BIO-TEK), 1,4-Dithio-DL-threitol (DTT) were purchased from Sigma-Aldrich Co. (St. Louis, USA). All other chemicals used were of the highest quality commercially available.

### Strains, plasmids and medium

*P. pastoris* GS115 and plasmid pPIC9K were purchased from the Invitrogen Company. Recombinant plasmid pPIC9K-*proRCL* and strain GS115/pPIC9K-*proRCL* were constructed by Yu *et al.* [21]. Yeast nutrient medium MD, MM, YPD-G418, YPD, BMMY and BMGY are prepared by means of “*P. pastoris* expression Kit” (*Pichia* Multi-Copy Expression Kit, version A, Invitrogen BV, The Netherlands).

### Design of Disulfide variants

Residues are numbered throughout this paper according to the three-dimensional models of *R. chinensis* lipase built by SWISS-MODEL. PyMOL was used for viewing and manipulating the *R. chinensis* lipase structures and models. Sites for insertion of disulfide bridges were selected using the program Disulfide by Design, which recognizes cysteine pairs that are in the proper orientation to form a disulfide bond [26].

### Three-dimensional structure simulation

A three-dimensional model of *R. chinensis* lipase in a close form was built by SWISS-MODEL protein automated modelling program [32] on the basis of crystal structure of lLGY (crystal structure of lipase II from *R. niveus* (RNL) solved with a resolution of 2.20 Å) [33]. The structure of *R. chinensis* lipase in a open form was modelled by SWISS-MODEL based on the three-dimensional crystallographic structure of 4TGL (crystal structure of the inhibited *R. miehei* lipase (RML) in an open form) [22]. The models were evaluated by Vadar (http://vadar.wishartlab.com/), which both showed good quality with a Z-score (standard deviation of the χ1 angles among all 3 (gauche-, gauche+, and trans) configurations) of 1.02. The amino acid sequence alignment between RCL, RNL and RML was shown in Fig. S1, which was 82.16% homology between RCL and RNL and 56.30% homology between RCL and RML, respectively.

### Plasmid construction

Oligonucleotide-directed mutations were incorporated into the lipase gene *proRCL* by overlap extension polymerase chain reaction (OE-PCR) [34], using plasmid pPIC9K-*proRCL* as template. Different synthetic oligonucleotides, carrying ‘mutated’ bases as listed (PHE 95-F/PHE95-R/PHE214-R/PHE214-F) in Table 2 were hybridized to the template DNAs and extended in an *in vitro* reaction. The restriction sites *Avr*II and *Not*I were incorporated into the forward and reverse primer sequence (RCLF/RCLR), respectively. The resulting PCR fragment, containing the mismatch sequences designed to change defined bases within the lipase gene *proRCL*, were subjected to double restriction and ligated into the respective sites of pPIC9K resulting in pPIC9K-proRCLCYS under the control of the methanol inducible alcohol oxidase 1 promoter (*P_AOX1_*) and fused in-frame with the α-factor secretion signal peptide of *Saccharomyces cerevisiae*. The mutations were verified by sequencing.

**Table 2:** Primers for plasmid pPIC9K-proRCLCYS construction.

Primers
PHE 95-F:
5′-ACTGACATGGTC*TGT*ACCTTTACTG-3′ (underlined ‘mutated’ bases for P95C)
PHE95-R:
5′-AATCAGTAAAGGT*ACA*GACCATGTCAG-3′ (underlined ‘mutated’ bases for P95C)
PHE214-F:
5′-CCTCCTCAAGCC*TGT*GGTTATCTTC-3′ (underlined ‘mutated’ bases for P214C)
PHE214-R:
5′-GAAGATAACC*ACA*GGCTTGAG-3′ (underlined ‘mutated’ bases for P214C)
RCLF:
5′-ATCGAACCTAGGGTTCCTGTTGCTGGTCATAAAG-3′ (underlined *Avr*II)
RCLR:
5′-CAGTGCGGCCGCTTACAAACAGCTTCCTTCGTT-3′ (underlined *Not*I)

### Transformation of *P. pastoris* and selection of recombinants

*P. pastoris* GS115 was transformed with *Bgl*II-linearized plasmids by electroporation, and selection of *His^+^* transformants was done on minimal dextrose medium (MD, Invitrogen BV) plate. The screening of geneticin resistant was performed on solid YPD-G418 medium. The insertion and methanol metabolization was checked by PCR. The PCR amplifications were carried out according to Invitrogen's recommendations with genomic DNA and primers complementary to the 5′ and 3′ region of the *AOX1* gene. Lipase secretion was assayed at 28°C on agar plate composed of minimal methanol medium (MM, Invitrogen BV) with 10 g/L olive oil and 1 mg/L fluorescent dye rhodamine B (MM-rhodamine) by the appearance of a fluorescent halo around colonies under UV light.

### Expression of lipase in *P. pastoris* in shake flasks

The *P. pastoris* transformants were cultured in 25 mL of buffered glycerol-complex medium (BMGY) shaken at 28°C and 250 rpm in 250-mL glass flasks. When cultures reached an OD_600_ of about 6, the cells were centrifuged and resuspended in 100 mL of buffered methanol-complex medium (BMMY) to an OD_600_ of 1.0, shaken at 28°C and 250 rpm in 500-mL glass flasks for 120 h. The cultures were supplemented with methanol (5 g/L) every 12 h to induce the expression of lipase.

### Lipase activity determination

Lipase activity was measured on emulsified *p*-nitrophenyl palmitate (pNPP) or *p*-nitrophenyl acetate (pNPA) according to Kordel *et al.* [35]. One enzyme unit was defined as the amount of enzyme releasing 1 µmol of *p*-nitrophenol per minute under the assay conditions. All the assays were done in triplicate, and significant differences (*p*<0.05) were measured.

### Lipase purification

The recombinant enzymes from the culture supernatant were purified after cultivation for 72 h. Cell free medium from the expression medium were concentrated and interchanged with 10 mM Tris-HCl buffer (pH 7.5) by ultrafiltration through a 10-kDa membrane (Millipore, USA). Then, the concentrated solution was loaded onto a SP-Sepharose column (Pharmacia, 1.6 cm×20 cm) equilibrated with 20 mM Tris-HCl buffer (pH 7.5), eluted with 0–0.5 M NaCl in the same buffer. Fractions containing lipase activity were pooled, concentrated and loaded on a Phenyl-sepharose 6 FF column (Pharmacia, 1.6 cm×20 cm) equilibrated in 50 mM Tris-HCl buffer (pH 7.5) containing 1.6 M ammonium sulfate. Lipase was then eluted in an ammonium sulfate concentration gradient decreasing from 1.6 to 0 M in 50 mM Tris-HCl buffer (pH 7.5) and 4 mL fractions were collected at a flow rate of 0.8 mL/min. Protein fractions were collected and assayed for protein concentration and lipase activity. Protein concentration was determined using Bradford assay. Bovine serum albumin (BSA) was used as a standard.

### Effect of temperature and pH on enzyme stability and activity

Optimal pH was determined by examining the activity of the enzyme at 40°C in the following buffers: 50 mM citrate (pH 5–6), 50 mM phosphate buffer (pH 7), 50 mM Tris-HCl (pH 8–8.5), and 50 mM glycine-NaOH (pH 9–10). Optimal temperature was determined by measuring the enzyme activity at optimal pH under various temperatures (20–60°C). Thermostability was determined by pre-incubating the purified enzyme for various intervals at temperature 60°C in optimal pH buffer and analyzing the residual activity.

### Kinetic parameters

The Michaelis-Menten kinetic parameters *k*_cat_ and *K*_m_ were calculated using pNPP or pNPA as substrate. Lineweaver-Burk plots were used to determine *k*_cat_ and *K_m_* parameters, assuming that the reactions followed a simple Michaelisk-Menten kinetics.

### Sodium dodecyl sulfate polyacrylamide gel electrophoresis

The protein was fractionated by SDS-PAGE system as described by Wu [36]. The sample (10 µl) was pretreated by mixing with 10 µl×2 SDS-PAGE loading buffer (20% (v/v) 0.5 M Tris-HCl (pH 6.8), 20% (v/v) glycerol, 4% (w/v) SDS, 0.1% (w/v) bromophenol blue) in a 0.5 ml centrifugal tube, 10 mM 1,4-dithiothreitol (DTT) under nonreducing condition was added. After vortexing for 5 s, the samples were incubated in 100°C water for 3 min, and then each sample aliquot (10 µl) was loaded to the sample well. Electrophoresis of the protein was performed on 15% separating gel with 3% stacking gel. The gels were kept at a constant voltage of 80 and 160 V at stacking and separating gel, respectively. Protein staining was completed by using a solution of coomassie brilliant blue.

### CD spectroscopy

Thermal unfolding was monitored by measurement of temperature-dependent circular dichroism (tCD) on a Chirascan spectropolarimeter at 220 nm with a 1 mm quartz cuvette. The spectra were obtained by heating protein solution over a temperature range from 20 to 70°C. Both proteins were at a concentration of 10 µM in 20 mM sodium phosphate buffer (pH 8.0).

### Biocatalysis of triglycerides and methanol for fatty acid methyl esters production

A typical substrate mixture was obtained by mixing 10 g of soy bean oil and methanol (*methanol* to oil molar ratio of 2∶1) containing saturated K_2_CO_3_ to keep water activity (*α*_w_) at 0.432. The reaction was carried out by adding 2000 U lipase into the substrate mixture in 20 mL screw-capped vials with teflon-lined septa incubated in a 40°C shaker. Samples (20 µL) were withdrawn at different intervals, and analyzed by GC described by He *et al.* [20]. Reactions were performed in triplicate.

## Supporting Information

Figure S1 **Alignment of the amino acid sequence of RCL, RNL and RML.**
(PDF)
