# Supplementary material for: Engineering a Disulfide Bond in the Lid Hinge Region of Rhizopus chinensis Lipase: Increased Thermostability and Altered Acyl Chain Length Specificity
Source: PLoS One. 2012 Oct 2;7(10):e46388. doi: 10.1371/journal.pone.0046388 (PMC3462767; doi:10.1371/journal.pone.0046388)
Supplement: Figure S1 — Alignment of the amino acid sequence of RCL, RNL and RML. (PDF) [file pone.0046388.s001.pdf]

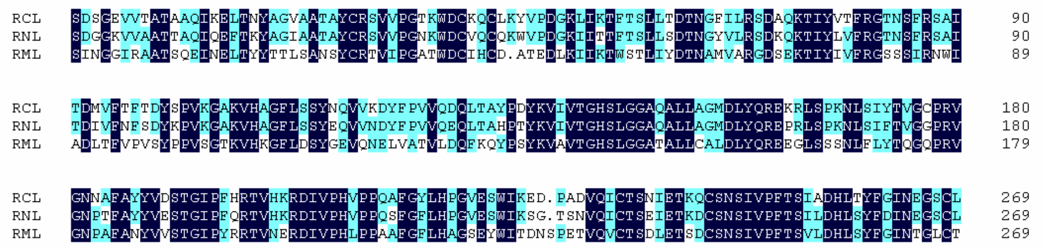

Figure S1. Alignment of the amino acid sequence of RCL, RNL and RML
